# Barriers Associated with the Uptake Ratio of Seasonal Flu Vaccine and Ways to Improve Influenza Vaccination Coverage among Young Health Care Workers in Poland

**DOI:** 10.3390/vaccines9050530

**Published:** 2021-05-20

**Authors:** Sylwia Kałucka, Izabela Grzegorczyk-Karolak

**Affiliations:** 1 Department of Coordinated Care, Medical University of Lodz, 90-251 Lodz, Poland; 2 Department of Biology and Pharmaceutical Botany, Medical University of Lodz, 90-151 Lodz, Poland; izabela.grzegorczyk@umed.lodz.pl

**Keywords:** barrier, influenza, influenza vaccine, midwifery, nursing, pharmacy, public health, vaccination coverage

## Abstract

Despite not being full-time health care workers, annual flu vaccination is nevertheless an important consideration for medical students. This study examined the reasons for refusing flu vaccination among medical students, a group characterized by low vaccination coverage, despite the fact that the flu vaccine is arguably the most effective way of preventing serious flu complications. A cross-sectional survey was performed of 1313 students at the Medical University of Lodz. The findings indicate that the main sites of vaccination were primary care centers, and main source of information about influenza vaccination (about 90% of cases) was the general practitioner (GP). The most common motivations for vaccination were a recommendation by the family doctor or the belief that it was an important factor for protection against influenza. Most students reported various adverse effects after vaccination, usually mild pain at the site of vaccination, malaise, or fever. The main reasons for rejecting influenza vaccination were the apparent low risk of disease, the need for annual vaccination, the need to pay for it, lack of time or opportunity, lack of vaccination promotion, negative attitudes toward the flu vaccine, or the belief that there are other methods of preventing flu. To increase long-term vaccine acceptance and increase the vaccination rate among medical students and qualified health care workers, there is a need to adapt the health system and to initiate ongoing promotion programs at university to raise consciousness, promote vaccinations, and develop clinical skills for immunization.

## 1. Introduction

Influenza is a highly infectious illness that can quickly lead to the development of local epidemics; it has a high risk of health complications and places a rapidly increasing burden on the health care system. The influenza virus itself, a member of the *Orthomyxoviridae*, is an RNA virus with a diameter of approximately 120 nm. The virus particle is composed of eight linked segments of single-stranded RNA forming the genome and a nucleoprotein capsid with glycoproteins embedded in lipoprotein envelope, such as hemagglutinin and neuraminidase. The viruses can be divided into four types, A, B, C, and D, based on antigen diversity; of these, only A and B infect humans [1].

An important tool for preventing influenza is the flu vaccine, which limits the spread of the epidemic and prevents serious complications [2,3]. Vaccinations are recommended for protecting high-risk groups such as pregnant women, seniors, and children below five years old from developing serious flu complications that can range from pneumonia, myocarditis, and brain encephalitis to multi-organ failure and even death [4]. According to the WHO, this high-risk group includes health care workers and medical students [5]. Although medical students are not full-time workers, their decision to receive an annual flu vaccination is a significant one.

Firstly, this group consists of young, healthy people who may be asymptomatic transmitters of the virus to their patients, other medical workers, and colleagues during classes in clinics, as well as to friends and family [6]. The students themselves are also at increased risk of infection because of their greater mobility, high activity dynamics, and relatively crowded living environment, including areas such as hospitals, university lectures, social gatherings, and sports clubs [7]. In addition, the students may only display mild flu symptoms, and therefore may not stay away from class [8]. Vaccination of health care staff is known to significantly reduce the risk of cross transmission and to limit the spread of epidemics [9,10]. 

Students, being young and feeling generally healthy, tend to perceive themselves as being at low risk of flu [11]. As such, it is difficult to persuade this group to vaccinate against influenza, especially when vaccination is optional, must be paid for, and should be repeated annually. Research conducted in the US indicates that compulsory vaccination programs are significantly more effective at achieving high vaccination rates than when it is only recommended [12].

In addition, students are often overlooked by the health care system and prevention programs. For example, in Germany, medical students are not included in the free on-site flu vaccinations for medical employees performed at medical institutions [13], despite also having regular contact with patients. In contrast, in Italy, the National Immunization Plan has recommended active and free flu vaccination to medical students since 2012 [1]. One such free vaccination policy increased vaccination coverage in Brazil by up to 75% in a short time [14].

It is clear that health care workers (HCWs) could play an essential role in promoting vaccination [15]. Therefore, being future HCWs, medical students, especially those in public health, should be more broadly engaged in the promotion of the seasonal influenza vaccine to society. However, to consciously promote vaccination, they also need to be vaccinated regularly.

Unfortunately, although about 80 years have passed since the invention of the first inactivated, monovalent influenza vaccine [16], flu vaccination coverage across the European Union remains at 44%, with Poland being significantly lower [17]. One reason for such low coverage is that most of the public have little knowledge about the flu vaccine and its health, economic, and social benefits [18]. In addition, taking the flu vaccine annually is often viewed as an inconvenience, even by health care workers [19]. As a result of such hesitancy among the public and the unpredictability of interest in vaccination, the government is typically unsure of how many vaccines to order, which represents a potentially serious threat to public health.

Although the World Health Organization and the Centers for Disease Control and Prevention strongly recommend annual vaccination for all health care staff, including physicians, nurses, and medical students [20,21], the annual flu vaccination rate among HCWs in many countries, including Poland, remains too low: the rate of regular flu vaccination remains between 3% and 25% among medicine students in Poland [22,23,24] and between 1% and 2.5% among public health, nursing, midwifery, and pharmacy students [25]. Similar low vaccination rates have been described for nursing students in Hong Kong (15.5%) [26], nursing students in Spain (5.3%) [27], and medical students in China (less than 10%) [7]. In contrast, vaccine coverage has been found to be over 70% among medical students in Canada in 2016 [28], 54% among medical students in Australia in 2014 [29] and in Italy in 2013/2014 [1], 43% among public health students in the US in 2015 [30], and between 40% and 60% among medical students in the US [13,30].

Although influenza vaccination uptake has been explored among medical students in Poland [22,23,24,25], little research has been carried out into attitudes toward vaccination among future HCWs. Therefore, the aim of the present study was to identify the sources of hesitancy regarding flu vaccination among medical students in Poland and to propose ways to increase uptake. To achieve this, the research assessed attitudes, doubts, and beliefs about the vaccine and factors influencing its uptake among a large group of medical students in four majors of medical science—namely, Nursing, Midwifery, Pharmacy, and Public Health, across all years of study at the Medical University of Lodz.

## 2. Materials and Methods

A cross-sectional study was performed among students studying four different majors of medical science—namely, Nursing, Midwifery, Pharmacy, and Public Health, at the Medical University of Lodz, Central Poland. Questionnaires were distributed to 1313 students aged 18 to 32 (mean age = 21.3 ± 1.6 years), and a total of 1188 were returned. The study took place between December 2019 and February 2020. The participants were informed by the main investigator about the purpose the study. They were also informed of the voluntary, confidential, and anonymous nature of the study and that the return of the completed questionnaire also indicated consent to participate in the study. The study was approved by the Medical Ethics Committee of the Medical University of Lodz (Poland) (ID: RNN/141/13/KB).

The final questionnaire contained three sections: (i) demographic information (age, major of study, year of study); (ii) immunization status (receipt of vaccination and number of times during the last three years); and (iii) a series of statements assessing attitudes and beliefs toward influenza vaccination and the circumstances surrounding influenza vaccination. This final part consisted of two closed questions for all vaccinated and unvaccinated participants: “*Can getting the flu cause complications such as pneumonia, bronchitis, myocarditis, meningitis, acute kidney failure, and other*?” and “*Do you think that flu vaccination significantly reduces the risk of these complications?”* Each question had three possible answers*: Yes, No,* or *I do not know*. The final part of the questionnaire also included one dichotomous (yes/no) question: “*Do you think there are other methods of preventing flu besides vaccination?”* with a request to list the methods if the answer was positive. 

The unvaccinated students were asked to give their reason for not being vaccinated. In addition, the vaccinated students were asked about their reason for being vaccinated and about the circumstances of the vaccination, such as *whether a medical examination was performed before vaccination*, *where the vaccination took place*, *how they found out about the vaccination,* and *what vaccine adverse events did they observe after vaccination*. 

The responses to the questionnaires were converted to electronic form by the first author; the output was double checked and analyzed by both authors independently. The content of the survey was analyzed in a classical way, grouping the most common student responses; before grouping, the key descriptors were extracted from the responses to open questions and labelled appropriately [31].

## 3. Results

### 3.1. Vaccination Status

Of the 1313 students at the Medical University of Lodz majoring in Nursing, Midwifery, Pharmacy, or Public Health in the academic year 2019/2020, 1188 students (90.5%) responded to the questionnaire. Of these, 48.9% of the Public Health students had been vaccinated in the last three years, although only 1.1% regularly and annually; 31% of Pharmacy students, including 2.5% annually; 30.7% of Nursing students, including 1.7% annually; and 25.1% of Midwifery students, including 2.4% annually. A detailed analysis of the flu vaccine coverage between different demographic groups during this period was described in our earlier study [25].

### 3.2. Influenza Risk Assessment and the Possibility of Reducing Risk by Vaccination

A key aim of the study was to determine the awareness of the complications associated with flu among the participants. It was found that more than half of the students of Nursing and even two-thirds of the remaining vaccinated medical students knew of the possible serious complications associated with catching the flu. Interestingly, such knowledge was also declared by between 49% (in the case of Public Health) to 80% (of Nursing) of those who had not been vaccinated (Table 1). Only a few to a dozen or so percent of the respondents did not think that catching the flu could be connected with serious complications. The majority of students believed that the flu vaccine could significantly reduce the risk of developing flu complications (Table 1). This was slightly more apparent among those who were vaccinated (82–98%) than among unvaccinated students of Pharmacy, Nursing, and Midwifery (82–88%). This value was significantly lower among unvaccinated Public Health students (57.8%). It follows that most of the medical students participating in the survey were aware of the consequences of catching the flu and knew that influenza vaccination is an effective method of preventing them.

### 3.3. The Reason for Rejecting or Accepting Influenza Vaccination among Medical Students

Among the vaccinated students from all four majors of study, the most common motivation for being vaccinated was a recommendation from their family doctor. This was true for both those who had been vaccinated only once (from 32.4% in Midwifery to 50% in Public Health) and those vaccinated regularly (from 29.7% in Pharmacy to 36.7% in Nursing) (Table 2).

Another common motivation was the awareness that the flu vaccine was the best way to prevent influenza, which was noted by 27–35% of students of all majors who vaccinated regularly. In addition, many of the students who had only been vaccinated once claimed that they had done so because it was free: between 20% and 26% of once-vaccinated Nursing, Midwifery, and Pharmacy students had received a free vaccination (Table 2). 

However, most of the students had not been vaccinated against the flu for the previous three years (Table 3). The most common reason was good health and the belief that there was a low risk of infection: 40% of the Nursing students, followed by 38.2% Pharmacy students, 35% Midwifery students, and 27.1% Public Health students. The second most common reason was the need to take the vaccine every year (Table 3): this response was given by at least one fifth of unvaccinated students from all majors. Other reasons included the fact that influenza vaccination was optional and required payment, and the lack of information on vaccination, including the lack of any influenza vaccination promotion or vaccination programs for students at university. A significant number of students also reported lack of time or a negative attitude toward the flu vaccine.

### 3.4. Access to Vaccination and Vaccination Circumstances

In Poland, a medical examination by a GP is obligatory before vaccination because only a doctor can qualify patients for vaccination and prepare a prescription for the purchase of a vaccine at a pharmacy. Therefore, most students in all four majors declared that they had been examined before the flu shot, regardless of whether they have been vaccinated once (from 81% to 90.5%) or regularly (from 73.9% to 93.9%) in the last three years (Table 4). Due to the medical health system, it is also not surprising that approximately 90% or more of vaccinations took place in GP practices (outpatient clinics). Some of the students were vaccinated in specialist clinics (25 students), and only few at home (15 students), in the workplace (5 students), or elsewhere (6 students), i.e., pharmacies, when the students were abroad on a scholarship (Table 4).

The vaccinated students from all four majors of study indicated the same three main sources of information on influenza vaccination (Table 5): family doctor, family members, and the mass media. However, of these, the family doctor was the most frequently chosen source of information, regardless of whether the participants had been vaccinated regularly or only once. Pharmacies, specialist doctors, or workplaces did not appear to play any significant role in promoting vaccination among young people in Poland.

The second most important source of information about influenza vaccination was family members; however, among Public Health students, this was a significantly less common source than GPs, both among those vaccinated once (26.7% compared with 53.3%) and those vaccinated regularly (23.4% compared with 48.4%) (Table 5).

### 3.5. Vaccine Adverse Events (VAE) of the Flu Vaccination

Around 80% of the regularly vaccinated Midwifery students reported vaccine adverse events (VAEs) after influenza vaccination. Among the students who had been vaccinated regularly in the last three years, the Nursing students reported nearly half the number of VAEs (40%) (Table 6). Among the students who had only been vaccinated once over the previous three years, 43% of Pharmacy students reported VAEs, compared with 52.2% of Nursing students, 61.1% of Midwifery students, and 66.7% of Public Health students.

Interestingly, although a fairly high number of negative side effects were reported, most of them concerned pain at the site of infection. Interestingly, this was more commonly felt/noticed by once-vaccinated respondents than those who had been regularly vaccinated, apart from Pharmacy students (Table 6). Regularly vaccinated Nursing and Midwifery students also commonly reported malaise, while Pharmacy students reported muscle pain, fever, and headache. 

### 3.6. Other Flu Prevention Methods

Between 65% and 76% of unvaccinated and 53% and 73% of vaccinated students in Pharmacy, Nursing, and Midwifery indicated that they could recommend other methods, apart from vaccination, that could help prevent flu (Table 7). A significantly lower positive response was recorded among unvaccinated (51.1%) and vaccinated Public Health students (23.3%). 

Among the students of Nursing, Midwifery, and Public Health who gave a positive answer, the following justifications predominated: leading a healthy lifestyle and using natural medicines supporting the immune system and vitamins (Table 7). As a healthy lifestyle, students indicated a healthy diet or physical activity, and most commonly mentioned raspberries, honey, garlic, onion, fish oil, vitamin C, or vitamin D as dietary supplements. They also recommended avoiding contact with sick people. In contrast, the Pharmacy students recommended maintaining proper hygiene rules (30–35%) followed by leading a healthy lifestyle (approximately 28%); following this, the vaccinated students recommended avoiding sick people, while the unvaccinated students recommended the use of medicines to increase the immune system.

## 4. Discussion

The most effective way to fight influenza is prophylaxis, and its most important element is the inactivated, intramuscular influenza vaccine. It reduces serious morbidity and mortality associated with influenza infection. About 80 years have passed since the invention of the first influenza vaccine; however, updated flu vaccines and annual vaccinations are still needed to cope with the high variability and mutation of flu viruses [1,32]. The seasonal flu vaccine is targeted against the strains that are believed to predominate during a particular year’s flu season, based on recommendations from the World Health Organization and the Working Party on Influenza of the European Medicines Agency. The flu vaccine is constantly being improved to make it more effective, but also safer. In the meantime, the vaccine has also been adapted from a monovalent form to quadrivalent forms [33]. Currently, across Europe, as well as in Poland, the flu vaccine is typically provided as a quadrivalent form, i.e., as inactivated subunit vaccines or split vaccines, which protect against four different viruses: two influenza A viruses and two influenza B viruses. However, due to too low annual vaccination coverage, the virus has not yet been eradicated, and it remains in the top ten most common global diseases in the 21st century. [1].

Our findings indicate and clarify the barriers to the adoption of influenza vaccination among four majors of medical students: Nursing, Midwifery, Pharmacy, and Public Health. These factors can be classified into three important thematic blocks: the organization of the health care system and vaccinations, including vaccine costs; the attitude toward influenza vaccination among participants; and promotion, education, and information about the flu vaccination among students at the Medical University. These issues are independent but also related.

Both the vaccinated and unvaccinated students were aware that the flu vaccine is effective at preventing the possible complications of influenza, such as pneumonia, bronchitis, or myocarditis (Table 1), and every third student stated that flu vaccination is the best method of preventing influenza (Table 2). Additionally, previous research has shown that Polish medical students have relatively high knowledge of the flu itself and about the flu vaccine [25,34]. However, this does not correspond to high vaccination coverage in this group.

The basis for efficient vaccination is the good organization of the health care system. In the Polish health care system, vaccinations are performed either by a family doctor or a nurse working in a primary health care facility who has completed a vaccination course. This system is typical of many European countries, where a family doctor leads the vaccination process [35]. In Poland, these recommendations are strictly implemented, as shown by the present responses regarding the circumstances of influenza vaccination (Table 4).

However, although this system may work well in vaccinating children, pregnant women, or seniors, it could also represent a barrier for medical students because it requires two appointments with their GP: first to get a prescription to buy a vaccine at a pharmacy, and then to allow medical examination by a doctor and vaccination by a nurse. This is a major inconvenience for students, who are often busy all day at university, and our present participants indicated in our survey the lack of time and possibility as a reason for no vaccination (Table 3). In addition, some medical students reported having their GP in their place of residence rather than their place of study; thus, they had no access to their GP from Monday to Friday when they were at university. 

Almost 90% of the vaccinated participants received the flu vaccination from their GP (Table 4), and 30% decided to vaccinate because it was recommended to them by their family doctor (Table 2). These results are alarming as, being younger, students tend to rarely visit their family doctor, which significantly reduces the likelihood of encountering the topic of vaccinations. Moreover, according to WHO recommendations for the Northern Hemisphere [36], influenza vaccination starts in September and is limited to the period of availability of vaccines at the pharmacies, which is usually between September and November: the majority of the population wants to be vaccinated before the increase in flu incidence, i.e., within three and four months. Therefore, limiting vaccination to a single place (sometimes far away from the place of daily residence), a single medical team (a GP and a nurse), and a short period of time presents a considerable obstacle to medical students.

Compared with Poland, where the majority of immunizations for flu are performed by a GP, influenza vaccinations in other countries are more commonly performed in pharmacies and supermarkets: they are visited by millions of people every week, they offer longer opening hours, and they do not require appointments [37,38]. In addition, vaccination coverage could be improved by extending the responsibility for providing immunization to other medical staff, such as midwives and pharmacists. Studies have identified higher immunization rates in societies that provide more favorable locations at which to receive vaccinations and that allow a wider range of medical workers (e.g., pharmacists and midwives) to perform them [39,40]. Such an approach, despite existing in other countries, would require structural changes to the Polish health care system, starting from training pharmacists, either during studies or afterward, adapting pharmacies to vaccination, and educating and convincing society about the possibility of vaccination against influenza. An easier and faster solution is to implement vaccinations in the medical facilities belonging to medical universities, for example during student clinical classes; this was found to be a successful solution in Germany [13]. However, in our study, fewer than 1% of participants reported that flu vaccination was promoted at university. In a Spanish study, the students themselves suggested that vaccination should be performed at the medical school itself, i.e., in the building where they receive their classes, rather than in a health care setting [31]. 

Vaccination against influenza should be free and mandatory for students starting clinical practice, which could solve the problems associated with promoting influenza prevention, lack of vaccine availability, getting appointments with family doctors, and convincing the unconvinced to vaccinate. Many of the participants reported that what finally persuaded them to receive a flu vaccination was that the service was free (Table 2). Therefore, another potential approach could be to remove the charge for vaccination [41,42]. Free vaccines may increase vaccine uptake among HCWs who can otherwise act as transmitters of the virus and increase the incidence of influenza, medical consultations, and hospitalization [43,44,45]. Currently, in Poland, the flu vaccine is not reimbursed by the state for HCWs, including medical university students. It is not possible to strengthen and improve vaccination rates in society without increasing vaccination coverage among medical staff [46]. Therefore, a stable and high level of flu vaccine uptake in society requires greater promotion of vaccination among HCWs [47].

On the other hand, free vaccination does not appear to be the ultimate solution to low vaccination coverage. One study in Slovakia found that the majority of students did not want to be vaccinated, even if they received the vaccine for free [48]. In this case, the main problem was the lack of knowledge and distrust toward vaccination. Additionally, the attitude toward the flu vaccination among our participants played a significant role in our findings. Although fewer than 10% of students in all majors directly indicated a negative attitude toward flu vaccination, this is one of the factors preventing the achievement of the WHO recommended immunization level in many countries around the world. This reluctance has been attributed to the need for annual vaccinations, the lack of faith in the effectiveness of the vaccine, and fear of its side effects. In order to reduce the fear of vaccination and the possible occurrence of VAE, and to maintain good knowledge and awareness of the need for influenza vaccination, training in vaccination should be introduced regularly throughout university studies. If students are not accustomed to getting vaccinated during their studies and do not perform vaccination, they are unlikely to become vaccinated after graduation and to promote vaccination among patients. It has been shown that participation in simulated vaccination clinics increases confidence among students, and this is believed to be associated with providing the knowledge regarding vaccination and the skills for safe vaccine administration [49].

Although vaccination commonly results in some vaccine adverse events (VAEs), most are mild. Similar results were also reported among the vaccinated students of our study. More than half of participants reported pain at the injection site, muscle aches, and/or malaise. Our findings show that even students who get vaccinated regularly do not become habituated and VAE can occur; as such, it is so important to inform the recipient about possible symptoms before/after vaccination. In addition, as even minor VAEs can cause complications in daily activities such as participating in classes, there should be the possibility for students to get one or two days free from university classes, without any obligation to make up the absence.

However, these VAEs do not outweigh the protective benefits of the flu vaccine. It is necessary to promote the social benefits of vaccination to medical students and other health professionals and to emphasize that the flu vaccine is an important part of preventive health care [50]. According to a large-scale study of more than 13,000 people from various European countries, improving understanding of vaccine effectiveness and safety would help to significantly increase vaccination rates [51]. HCWs are not only themselves at high risk of developing disease and serious complications (including death) but can also transmit the virus. Data in the literature report that vaccination for medical staff was associated with a 40% reduction in mortality among senior care home residents [52] and incidents associated with influenza-like diseases [53]. This would be a good approach for encouraging vaccination among medical students: a significant factor in their low motivation for vaccination was their belief that they personally were at low risk of catching flu [26,54]. Moreover, every third student of our survey who reported not being vaccinated justified their hesitancy by claiming that they do not get sick (Nursing 39.8%, Midwifery 35%, Pharmacy 38.2%, Public Health 27.1%). 

Medical students should also be alerted to the fact that as HCWs they are also the best role models to be followed by the rest of the general population. In Poland, for several years seasonal influenza vaccination uptake has remained low both among HCWs including doctors (approximately 22%) and nurses (5–10%) [55] and in the general population (9.5%); below the EU average [56]. Therefore, the lack of importance attached to vaccination by HCWs, including medical students, reflects the low vaccination coverage among the general public, as in Poland; in contrast, a high vaccination rate among medical students and qualified HCWs, e.g., 73% coverage in the US (82% among doctors and 62% among nurses) has a positive effect on the general population [46]. Similar findings have been confirmed in other studies [29,42,57].

The present group of participants also cited the need for repeated vaccination every year with new updates to cope with new mutations [33] as a reason for rejecting vaccination. The vaccination system in Poland also seems to suffer from a lack of promotion and information regarding flu vaccination programs (Table 5). Only a few students reported that mass media recommendation or advertisement played a role in their decision to get vaccinated (Table 2). Hence, non-vaccinated students have little possibility to learn about vaccination programs, and students who have a negative attitude to vaccination (Nursing 10.2%, Midwifery 4,5%, Pharmacy 7.7%, Public Health 6.8%) do not have the opportunity to change their point of view; more than 50% of students, vaccinated and non-vaccinated, reported looking for other, more easily available, alternatives for flu prevention (Table 7). Interestingly, in all majors, the vaccinated students were less likely to indicate such methods than those who were not vaccinated. Nursing, Midwifery, and Public Health students recommended a healthy lifestyle and supplementation with vitamins C and D, as well as the use of natural products which, in their opinion, support the immune system, among the two main factors for preventing flu. Pharmacy students also indicated the observance of proper hygiene rules, such as washing hands. Although the promotion of a healthy lifestyle is beneficial in the prevention of infectious and socially transmitted diseases, it is of possible concern that the participants considered natural products and proper hygiene to be equivalent, or even a replacement for the influenza vaccine, rather than as a means of supporting other health-promoting activities.

## 5. Conclusions

Our findings indicate that a number of barriers exist to improving vaccination coverage at Medical University among students majoring in Nursing, Midwifery, Pharmacy, or Public Health. Most of all, many different logistical and organizational barriers influence the uptake of the flu vaccine, and these should be considered for revision to ensure greater access to vaccination. As the vaccine is optional, must be paid for, and there are no organizational structures enabling easy access, medical students have many excuses for not getting vaccinated, such as not having enough time, schedule possibilities, or money; they also tend to possess a strong belief in their own good health and a low risk of infection. 

There is hence a need for systemic and organizational changes in the health care system, which are essential for improving influenza vaccination coverage among future health care workers and society in general. To significantly increase influenza vaccination coverage in the general population in Poland, there is also a need for vaccination promotion activities, such as providing convenient locations for flu vaccination, e.g., workplaces for staff and on campus for students, providing financial support for vaccination, setting aside vaccination time, offering vaccination classes that improve knowledge regarding vaccines, and allowing other groups of HCWs (e.g., pharmacists, midwives, public health workers) to perform influenza vaccinations. Such initiatives have been found to have positive effects in other countries. It is important that medical students not be overlooked in flu vaccination campaigns and that a culture of vaccine promotion should be fostered in medical universities; this could include extra bonuses and incentives for the vaccinated. Such systems may well enhance vaccine uptake among health care workers, which will indirectly translate into increased vaccination coverage throughout society.

## Figures and Tables

**Table 1:** Influenza risk assessment and the possibility of reducing risk by vaccination, according to student major.

Influenza Complication	Nursing		Midwifery		Pharmacy		Public Health	
	Vaccinated 137 (30.7%)	Unvaccinated 309 (69.3%)	Vaccinated 42 (25.1%)	Unvaccinated 125 (74.9%)	Vaccinated 135 (31%)	Unvaccinated 301 (69%)	Vaccinated 43 (48.9%)	Unvaccinated 45 (51.5%)
**Complications after flu**								
Yes	74 (54%)	221 (71.5%)	26 (61.9%)	100 (80%)	83 (61.5%)	246 (81.7%)	30 (69.8%)	22 (48.9%)
No	23 (16.8%)	36 (11.7%)	7 (16.7%)	11 (8.8%)	25 (18.5%)	35 (11.6%)	1 (2.3%)	7 (15.6%)
I do not know	40 (29.2%)	52 (16.8)	9 (21.4%)	14 (11.2%)	27 (20%)	20 (6.7%)	12 (27.9%)	16 (35.6%)
**Influenza vaccination reduces the risk of complications from influenza**								
Yes	130 (94.9%)	271 (87.7%)	41 (97.6%)	103 (82.4%)	111 (82.2%)	266 (88.4%)	36 (83.7%)	26 (57.8%)
No	3 (2.2%)	10 (3.2%)	0 (0.0%)	6 (4.8%)	11 (8.1%)	12 (4.0%)	3 (7.0%)	5 (11.1%)
I do not know	6 (4.4%)	28 (9.1%)	1 (2.4%)	16 (12.8%)	13 (9.6%)	23 (7.6%)	4 (9.3%)	14 (31.1%)

**Table 2:** Stated reason for vaccination among students, according to major.

Vaccination Reason	Nursing		Midwifery		Pharmacy		Public Health	
	Once	Regularly	Once	Regularly	Once	Regularly	Once	Regularly
Family doctor recommendation	45 (34.9%)	18 (36.7%)	11 (32.4%)	12 (30%)	27 (38%)	43 (29.7%)	5 (50%)	23 (36.5%)
Possibility of free vaccination	26 (20.2%)	8 (16.3%)	9 (26.5%)	9 (22.5%)	18 (25.4%)	28 (19.3%)	0 (0.0%)	17 (27%)
The best method of flu prevention	27 (20.9%)	14 (28.6%)	9 (26.5%)	13 (32.5%)	14 (19.7%)	51 (35.2%)	5 (50%)	17 (27%)
Friend’s recommendation	12 (9.3%)	1 (2%)	2 (5.9%)	2 (5%)	1 (1.4%)	9 (6.2%)	0 (0.0%)	1 (1.6%)
Advertisement/leaflets at the clinic	1 (0.8%)	1 (2%)	0 (0.0%)	1 (2.5%)	0 (0.0%)	6 (4.1%)	0 (0.0%)	2 (3.2%)
Recommendation in the mass media	0 (0.0%)	5 (10.2%)	1 (2.9%)	0 (0.0%)	0 (0.0%)	3 (2.1%)	0 (0.0%)	0 (0.0%)
Accidentally	3 (2.3%)	0 (0.0%)	0 (0.0%)	3 (7.5%)	9 (12.7%)	1 (0.7%)	0 (0.0%)	1 (1.6%)
Other	15 (11.6%)	2 (4.1%)	2 (5.9%)	0 (0.0%)	2 (2.8%)	4 (2.8%)	0 (0.0%)	2 (3.2%)

**Table 3:** Reasons for not receiving a flu vaccination, according to student major.

Reason for Non-Vaccination	Nursing	Midwifery	Pharmacy	Public Heath
Annual vaccination required	70 (20.9%)	37 (23.6%)	79 (20.9%)	14 (23.6%)
No need, I do not get sick	133 (39.8%)	55 (35%)	144 (38.2%)	16 (27.1%)
No occasion, no time	32 (9.6%)	5 (3.2%)	25 (6.7%)	3 (5.1%)
Negative attitudes toward the flu vaccine	34 (10.2%)	7 (4.5%)	29 (7.7%)	4 (6.8%)
No information	16 (4.8%)	26 (16.6%)	50 (13.3%)	7 (11.9%)
Parental decision	4 (1.2%)	5 (3.2%)	3 (0.8%)	3 (5.1%)
Optional, paid vaccination	45 (13.5%)	18 (11.4%)	42 (11.1%)	7 (11.9%)
Other	0 (0.0%)	4 (2.5%)	5 (1.3%)	5 (8.4%)

**Table 4:** Qualification for vaccination and its location, according to student major and frequency of vaccination.

Vaccination Circumstances	Nursing		Midwifery		Pharmacy		Public Health	
	Once	Regularly	Once	Regularly	Once	Regularly	Once	Regularly
**Medical examination before vaccination**								
Yes	76 (86.4%)	34 (73.9%)	17 (81%)	23 (92%)	50 (90.9%)	73 (92.4%)	9 (90%)	31 (93.9%)
No	12 (13.6%)	12 (26.1%)	4 (19%)	2 (8%)	5 (9.1%)	6 (7.6%)	1 (10%)	2 (6.1%)
**Vaccination location**								
Family doctor/GP	81 (89%)	41 (83.7%)	20 (90.9%)	24 (96%)	42 (82.4%)	65 (81.25%)	10 (100%)	28 (82.4%)
Specialist clinic	10 (11%)	3 (6.1%)	1 (4.55%)	0 (0.0%)	2 (3.9%)	4 (5%)	0 (0.0%)	5 (14.7%)
Home	0 (0.0%)	5 (10.2%)	0 (0.0%)	0 (0.0%)	0 (0.0%)	9 (11.25%)	0 (0.0%)	1 (2.9%)
Workplace	0 (0.0%)	0 (0.0%)	0 (0.0%)	0 (0.0%)	5 (9.8%)	0 (0.0%)	0 (0.0%)	0 (0.0%)
Other	0 (0%)	0 (0%)	1 (4.55%)	1 (4%)	2 (3.9%)	2 (2.5%)	0 (0.0%)	0 (0.0%)

**Table 5:** The source of information about vaccination reported by students, according to major.

Information about Vaccination	Nursing		Midwifery		Pharmacy		Public Health	
	Once	Regularly	Once	Regularly	Once	Regularly	Once	Regularly
Family doctor	49 (38%)	22 (36.7%)	13 (37.1%)	19 (38.8%)	34 (38.2%)	51 (43.2%)	8 (53.3%)	31 (48.4%)
Medical specialist	4 (3.1%)	3 (5%)	2 (5.7%)	2 (4.1%)	2 (2.2%)	7 (5.9%)	0 (0.0%)	5 (7.8%)
Pharmacist	2 (1.6%)	3 (5%)	0 (0.0%)	0 (0.0%)	2 (2.2%)	7 (5.9%)	0 (0.0%)	1 (1.6%)
From television	16 (12.4%)	2 (3.3%)	5 (14.3%)	7 (14.3%)	5 (5.6%)	6 (5.1%)	1 (6.7%)	6 (9.4%)
From the radio	8 (6.2%)	1 (1.7%)	1 (2.9%)	1 (2%)	4 (4.5%)	0 (0.0%)	0 (0.0%)	2 (3.1%)
From the press	4 (3.1%)	1 (1.7%)	1 (2.9%)	1 (2%)	0 (0.0%)	0 (0.0%)	0 (0.0%)	4 (6.3%)
From the workplace	3 (2.3%)	0 (0.0%)	0 (0.0%)	1 (2%)	4 (4.5%)	0 (0.0%)	0 (0.0%)	0 (0.0%)
From family members	37 (28.7%)	25 (41.7%)	11 (31.4%)	16 (32.7%)	31 (34.6%)	39 (33.1%)	4 (26.7%)	15 (23.4%)
From friends /neighbors	4 (3.1%)	0 (0.0%)	2 (5.7%)	2 (4.1%)	7 (7.9%)	6 (5.1%)	2 (13.3%)	0 (0.0%)
Other	2 (1.6%)	3 (5%)	0 (0.0%)	0 (0.0%)	0 (0.0%)	2 (1.7%)	0 (0.0%)	0 (0.0%)

**Table 6:** Vaccine adverse events reported following flu vaccination, according to major and frequency of vaccination.

VAEs	Nursing		Midwifery		Pharmacy		Public Health	
	Once	Regularly	Once	Regularly	Once	Regularly	Once	Regularly
None	43 (47.8%)	27 (60%)	7 (38.9%)	4 (20%)	31 (56.4%)	26 (32.5%)	3 (33.3%)	9 (26.5%)
Yes	47 (52.2%)	18 (40%)	11 (61.1%)	16 (80%)	24 (43.6%)	54 (67.5%)	6 (66.7%)	25 (73.5%)
**What?**								
Pain at the injection site	28 (45.1%)	8 (33.3%)	9 (64.3%)	12 (48%)	9 (27.3%)	26 (40.6%)	4 (57.1%)	15 (24.9%)
Fever	6 (9.7%)	4 (16.7%)	1 (7.1%)	3 (12%)	4 (12.1%)	5 (7.8%)	1 (14.3%)	3 (5.9%)
Malaise	6 (9.7%)	6 (25%)	2 (14.3%)	6 (24%)	6 (18.2%)	7 (10.9%)	1 (14.3%)	1 (2%)
Muscle pain	11 (18.3%)	3 (12.5%)	1 (7.1%)	3 (12%)	3 (9.1%)	7 (10.9%)	1 (14.3%)	5 (9.8%)
Headache	3 (4.8%)	0 (0.0%)	1 (7.1%)	1 (4%)	3 (9.1%)	1 (1.6%)	0 (0.0%)	1 (2%)
Allergic reaction	0 (0.0%)	1 (4.2%)	0 (0.0%)	0 (0.0%)	0 (0.0%)	0 (0.0%)	0 (0.0%)	1 (2%)
Other	8 (12.9%)	2 (8.3%)	0 (0.0%)	0 (0.0%)	8 (24.2%)	18 (28.1%)	0 (0.0%)	25 (49%)

**Table 7:** Other methods for preventing flu reported by students, according to major and vaccination status.

Other Flu Prevention Methods	Nursing		Midwifery		Pharmacy		Public Health	
	Vaccinated	Unvaccinated	Vaccinated	Unvaccinated	Vaccinated	Unvaccinated	Vaccinated	Unvaccinated
No	65 (47.4%)	108 (35%)	18 (42.9%)	35 (28%)	36 (26.7%)	73 (24.3%)	33 (76.7%)	22 (48.9%)
Yes	72 (52.6%)	201 (65)	24 (57.1%)	90 (72%)	99 (73.3%)	228 (75.7%)	10 (23.3%)	23 (51.1%)
**What?**								
Healthy lifestyle	32 (28.1%)	96 (32.5%)	15 (37.5%)	31 (29.2%)	33 (27.3%)	89 (28.9%)	5 (35.7%)	4 (19%)
Medicines that increase immunity system	43 (38.7%)	81 (27.5%)	14 (35%)	31 (29.2%)	10 (8.3%)	43 (14%)	8 (57.1%)	8 (38.1%)
Avoiding sick people	13 (11.7%)	51 (17.3%)	5 (12.5%)	17 (16%)	17 (14%)	36 (11.7%)	1 (7.1%)	4 (19%)
Proper hygiene rules	3 (2.7%)	19 (6.4%)	0 (0.0%)	8 (7.5%)	42 (34.7%)	95 (30.8%)	0 (0.0%)	1 (4.8%)
Weather-appropriate outfit	8 (7.2%)	18 (6.1%)	3 (7.5%)	5 (4.7%)	6 (5%)	14 (4.5%)	0 (0.0%)	4 (19%)
Others	12 (10.8%)	30 (10.2%)	3 (7.5%)	14 (13.2%)	13 (10.7%)	31 (10.1%)	0 (0.0%)	0 (0.0%)

## Data Availability

The data presented in this study are available on request from the corresponding author.
